# Structurally Stable, High-Strength Graphene Oxide/Carbon Nanotube/Epoxy Resin Aerogels as Three-Dimensional Skeletal Precursors for Wave-Absorbing Materials

**DOI:** 10.3390/gels8100618

**Published:** 2022-09-28

**Authors:** Lina Zhang, Guojun Song, Zetian Zhao, Lichun Ma, Hui Xu, Guanglei Wu, Yinghu Song, Yinuo Liu, Lihan Qiu, Xiaoru Li

**Affiliations:** Institute of Polymer Materials, School of Material Science and Engineering, Qingdao University, Qingdao 266071, China

**Keywords:** graphene oxide aerogel, carbon nanotubes, epoxy resin, mechanical property, microwave absorption

## Abstract

Three-dimensional (3D) graphene oxide aerogel (GOA) is one of the best fillers for composites for microwave absorption. However, its further development has been hindered by the poor mechanical properties. Methodology to improve the mechanical properties of the aerogel remains an urgent challenge. Herein, graphene oxide/carbon nanotube/epoxy resin composite aerogel (GCEA) was successfully prepared by a facile method. The results showed that the prepared GCEA with the hierarchical and 3D cross-linked structures exhibited excellent compression performance, structural and thermal stability, high hydrophilicity, and microwave absorption. The prepared GCEA recovered from multiple large strain cycles without significant permanent deformation. The minimum reflection loss (RL) was −39.60 dB and the maximum effective absorption bandwidth (EAB) was 2.48 GHz. The development of the enhanced GO aerogels will offer a new approach to the preparation of 3D microwave-absorbing skeletal materials with good mechanical properties.

## 1. Introduction

While the development of electronic information technology has brought convenience and guarantees to human life and national defense security, this has been accompanied by various security issues, such as electromagnetic interference, electromagnetic (EM) radiation, and information leakage. A substantial amount of work has been done so far in developing “thin, light, wide, strong” microwave-absorbing (MA) materials to eliminate electromagnetic radiation [1,2]. Popular absorbing materials include zero-dimensional (0D) nanoparticles, one-dimensional (1D) nanotube and nanowire, two-dimensional (2D) MXene and graphene, etc. [3,4,5]. In an attempt to better meet impedance matching and attenuation characteristic, it is efficient to combine dielectric loss materials and magnetic loss materials to prepare MA materials, and the MA properties can be adjusted by adjusting the components [6]. In addition, the structure also influences the performance of the MA material, and three-dimensional (3D) network structures have been believed to extend the reflection/scattering path of incident waves and improve the absorbing properties of materials [7,8,9]. Lightweight foams and alumina templates have been used as 3D skeletons and coated with functional particles to prepare lightweight and efficient MA materials [10,11,12]. However, Alumina templates have been very cumbersome to handle, while the foams have negligible MA properties. If these skeletons could be replaced by MA materials with 3D structures, the MA properties of the composite would be further enhanced. Therefore, it is very important to design a 3D skeleton structure with microwave absorption and structural stability.

In recent years, the preparation and application of GO aerogels (GOA) has attracted a lot of attention due to their many unique properties, such as light weight, large specific surface area, and network interconnection structure. However, their unstable structures are well known [13,14,15]. Carbon nanotubes (CNTs) bridging adjacent GO via π-π bonds could significantly improve the mechanical strength of GOA [16,17,18]. Furthermore, CNTs have been often used as dielectric loss MA materials due to their excellent electrical conductivity and outstanding specific surface area [19,20].

Another approach to improve the mechanical strength of GOA is to increase crosslinking sites. Common candidate polymers for this approach include phenolic resins [21],polyvinyl alcohol (PVA) [22,23], chitosan [24], and cellulose [25,26]. In particular, waterborne epoxy resin is rich in amino and epoxy groups, and has been widely studied for its superior mechanical properties, chemical stability, and environmental friendliness. The introduction of the epoxy resin will be expected to add more functional groups and form more bonds with GO to improve the cross-linking inside the composite and increase the mechanical strength of the composite.

In this work, GO/FCNTs/epoxy composite aerogel (GCEA) with 3D structure was prepared by simple hydrothermal and freeze-drying methods. Epoxy resin increased the cross-linking points and FCNTs bridged the GO sheets, which enhanced the mechanical properties of the composite aerogel through synergistic effects. 3D structure effectively improved the absorption of microwave, and the introduction of FCNTs increased the absorption effect of microwave. The prepared 3D GCEA in this word had excellent structural stability, thermal stability, and mechanical strength, which can be used as the microwave absorption base material or as the reinforced skeleton of composite materials.

## 2. Results and Discussion

### 2.1. Elemental Analysis

**Figure 1:**
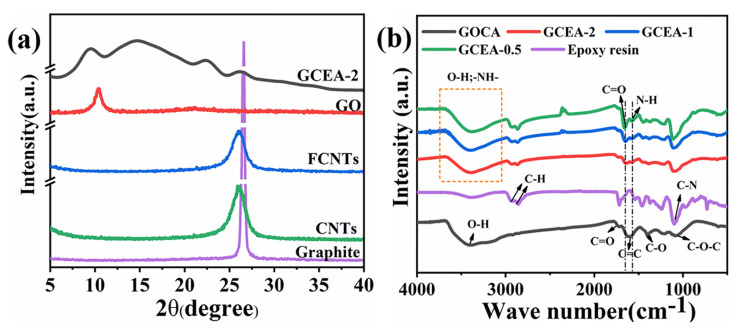
The XRD patterns (**a**) and FT-IR spectra (**b**) of the various samples (Color figure can be viewed online).

Figure 1a shows the XRD patterns of aerogels. The diffraction peak representing (001) plane of GO shifted from 10.2° to 9.5° in GCEA-2, and the interlayer spacing increased from 0.86 nm to 9.3 nm. As seen in Figure S1, the interlayer spacing increased with the epoxy content increasing, indicating that TETA was covalently bonded onto the GO to increase the layer spacing. The peak of GCEA around 26° belonged to the typical peak of FCNTs, and the broad peak in the 14~24.9° range may relate to the interaction between GO/FCNTs and epoxy resin.

FT-IR spectra were carried out to reveal the chemical compositions of the GOCA and GCEA and epoxy resin. As shown in Figure 1b, the feature peaks of GOCA at 3386, 1720, 1620, 1405, and 1070 cm^−1^ were due to the vibrations of the carboxyl O-H and C=O, the C=C stretching vibrations of the aromatic rings, and vibrations of the epoxide C-O and C-O-C, respectively [27]. For epoxy resin, the peaks at 2933 and 2855 cm^−1^ were attributed to stretching vibrations of C-H in the CH_2_ groups, and the peak at 1096 cm^−1^ was attributed to stretching vibrations of C-N in the amino groups. In particular, for GCEA, the new peak appeared at 1569 cm^−1^ due to the N-H stretching vibration [28]. At the same time, the peak of the carboxylic C=O stretching vibration shifted from 1720 cm^−1^ to 1654 cm^−1^ for the GCEA, suggesting that the TETA reacted with the GO and FCNTs to form amide bonds. In addition, the intensities of the new peaks increased with the increase of content of epoxy resin, indicating that the amount of grafting onto the carrier increased with the increase of cross-linker content.

**Figure 2:**
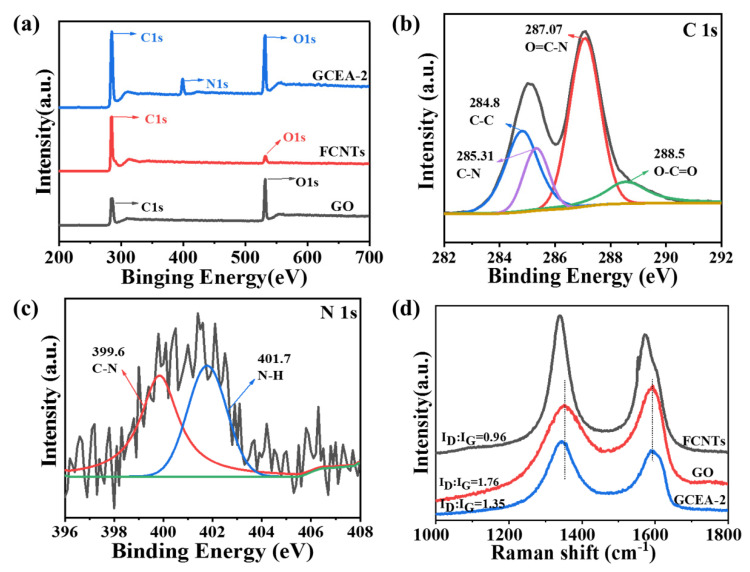
(**a**) XPS spectra, (**b**) the C1s and (**c**) N1s of the GCEA-2, (**d**) Raman spectra of GO, FCNTs, GCEA-2 (Color figure can be viewed online).

The surface chemical states of samples were identified by the high-resolution XPS measurement. As shown in Figure 2, the peaks of C 1s and O 1s were observed near 285.8 and 532.6 eV, respectively (Figure 2a), while the successful attachment of TETA to the GO was demonstrated by an additional N 1s peak at 399.0 eV for GCEA-2 (Figure 2a). The peaks at 284.8, 285.31, 287.07, and 288.5 eV in Figure 2b were respectively ascribed to the C-C, C-N [27], O=C-N, and O-C=O [29] groups. Meanwhile, the deconvolution N 1s spectrum of the GCEA-2 in Figure 2c showed two distinct peaks at 399.6 and 401.7 eV due to C-N and N-H, respectively. Thereby, this confirmed the chemical bonding of the epoxy resin onto the GO sheets. 

The Raman data are shown in Figure 2d and Figure S1c,d. Band D at 1342 cm^−1^ is generated by the edge and sp^3^ hybridized carbon bond defects. The G band at 1592 cm^−1^ relates to the sp^2^ carbon domain, where the integrated intensity ratio (*I_D_*/*I_G_*) between D and G band reflects the defect degree of carbon material [30] D band transferred from 1354 cm^−1^ in GOCA to 1341 cm^−1^ in GCEA-2, indicating a strong interaction between GO and TETA [29]. The *I_D_*/*I_G_* ratio of graphene oxide decreased from 1.76 to 1.35. Compared with GO, the defects of GCEA were reduced, and the grafted molecules replaced part of the sp^3^ carbon sites of graphene oxide to generate more sp^2^ carbon forms, which made the defects get repaired [30]. With the increase of cross-linker content, I*_D_*/*I_G_* slightly increased, which may be attributed to the epoxy coating on the GO sheet, leading to an increase of the disorder.

### 2.2. Morphological Characterization

**Figure 3:**
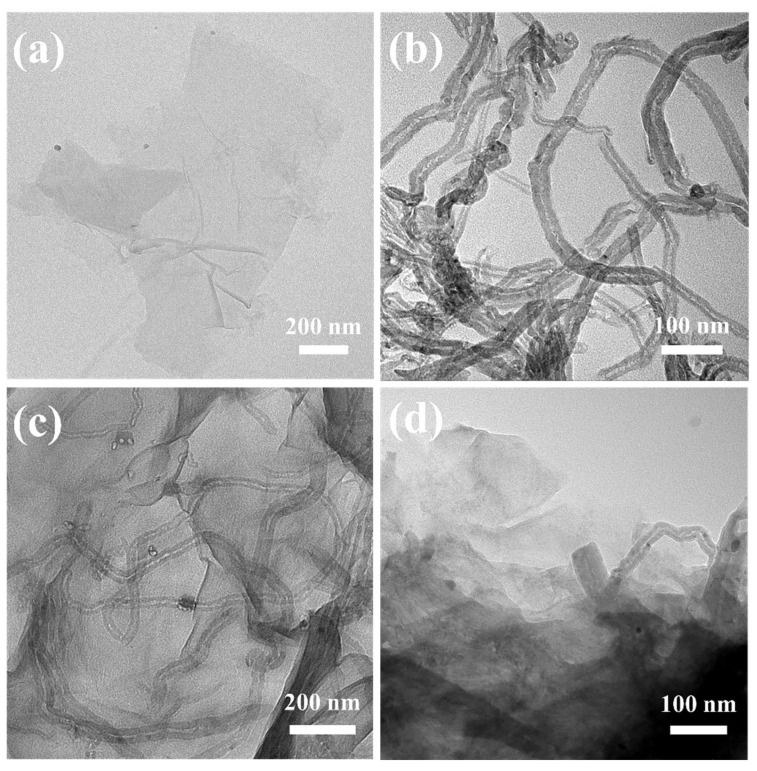
The TEM images of (**a**) the pristine GO, (**b**) the FCNTs, (**c**) the GO/FCNTs dispersion, and (**d**) GCEA-2 (Color figure can be viewed online).

**Figure 4:**
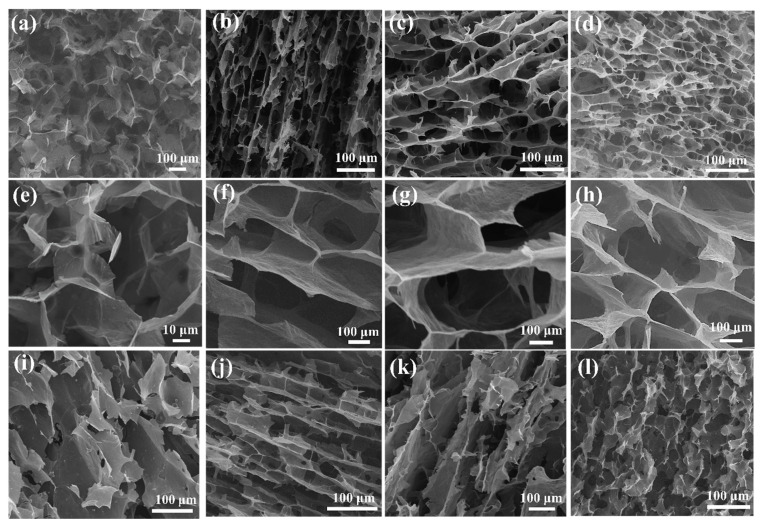
The SEM images of (**a**,**e**,**i**) the GOCA, (**b**,**f**,**j**) the GCEA-2, (**c**,**g**,**k**) the GCEA-1, and (**d**,**h**,**l**) GCEA-0.5 (Color figure can be viewed online).

The TEM images of the FCNTs, GO, and GO/FCNTs dispersion are presented in Figure 3. GO has a wrinkled nano-sheet structure (Figure 3a), and FCNTs nanotubes with diameters of 20~40 nm (Figure 3b) were uniformly dispersed among the transparent wrinkled GO sheets (Figure 3c). The GO sheets and FCNTs became significantly thicker after the addition of epoxy resin (Figure 3d). The SEM images (Figure 4) revealed that the pore size of all aerogels was in the micron level, and the pore walls of the GCEA were more complete and homogeneous than those of the GOCA and tended to be anisotropic. This was attributed to the successful grafting of TETA onto the GO surface, resulting in more cross-linking points and weakening the effect of ice crystals.

### 2.3. Hydrophilic and Structural Stability

**Figure 5:**
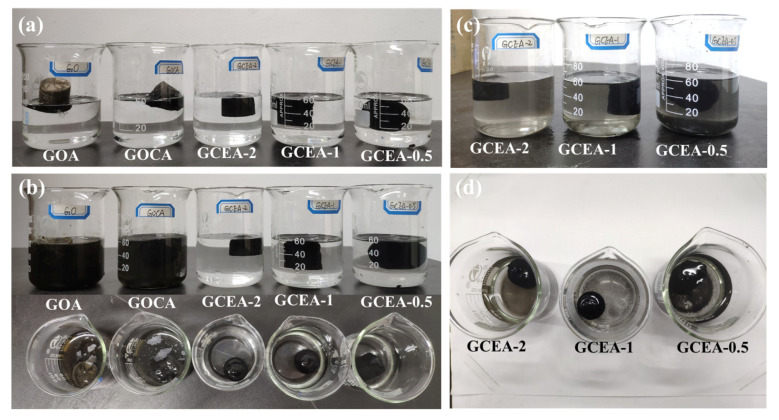
The digital images of GOA, GOCA, and GCEA immersed in water, (**a**) 0 min, (**b**) ultrasonic treatment for 1 min (side and top view), (**c**,**d**) the GCEA in ultrasonic treatment for 30 min (Color figure can be viewed online).

To evaluate the structural stability of the samples, they were placed in an ultrasonic environment. Compared with the GOA and GOCA, the instantaneous sedimentation rate of the GCEA in water was faster (Figure 5a). After 1 min of ultrasonic treatment at 53 kHz (Figure 5b), the structures of the GOA and GOCA were damaged, however, the GCEA remained intact. The structure of the GCEA remained intact even after 30 min of ultrasonic treatment (Figure 5c,d). The above experimental phenomena proved that GCEA has more hydrophilic and more stable structure than that of GOA and GOCA.

### 2.4. Mechanical Properties 

**Figure 6:**
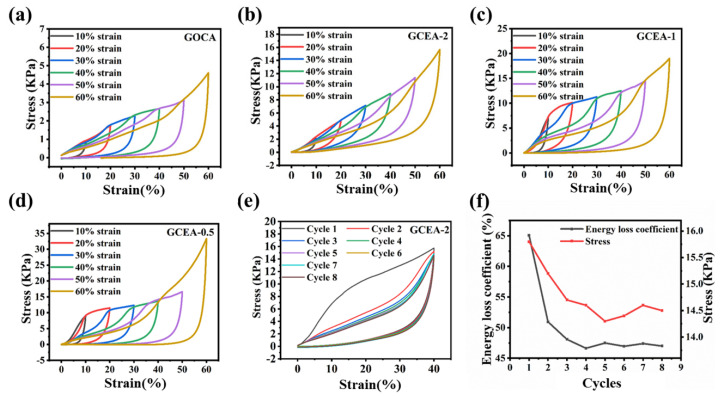
(**a**–**d**) The stress–strain curves of GOCA, GCEA aerogel at different maximum strains, (**e**) stress–strain curves of GCEA-2 at an extreme strain of 40% for 8 cycles, (**f**) energy loss coefficient and stress of carbon aerogels after repeated compression at a strain of 40% (Color figure can be viewed online).

The cyclic compression stress–strain curves of the GOCA and GCEA are presented in Figure 6a–d and Figure S2. Here, three elastic regions were identified during the loading process and defined by the shape variable (ε) [31]. They were linear elastic region, yield region, and dense region, respectively. The ε value was less than 10% in the linear elastic region, 10% < ε < 50% in the yield region, and ε > 50% in the dense zone. The 3D structure was destroyed in the dense zone, and the stress increased due to the decrease in pore volume. Compared with the GOCA, the compressive strength of the GCEA was significantly enhanced. Among these samples, GCEA-2 showed good compression performance with minimum compression loss per cycle. The cyclic stress–strain curves of the various samples at strains of up to 40% are shown in Figure 6e,f and Figure S3. The GCEA-2 exhibited the strongest cyclic recovery ability, with the first cycle showing the maximum stress and the highest hysteresis curve, which corresponded to typical viscoelastic behavior [32]. The stress–strain curve was essentially unchanged up until the 8th cycle, and the overlapping unloading curve indicated that the material could recover its original height. The calculated loss factor was 65.08%, which was suitable for energy-absorbing materials, and the multi-cycle loss factor and stress decrease were attributed to the bending of the hole walls. The results in Figure S2 clearly indicated that the GCEA-3 had similar mechanical properties to those of the GCEA-2, but with a lower strength due to the low epoxy content. The mechanical properties of the GCEA-0.33 were increased due to the increase in epoxy content, but its compressibility was greatly reduced due to its brittle nature.

### 2.5. Thermal Stability Analysis

**Figure 7:**
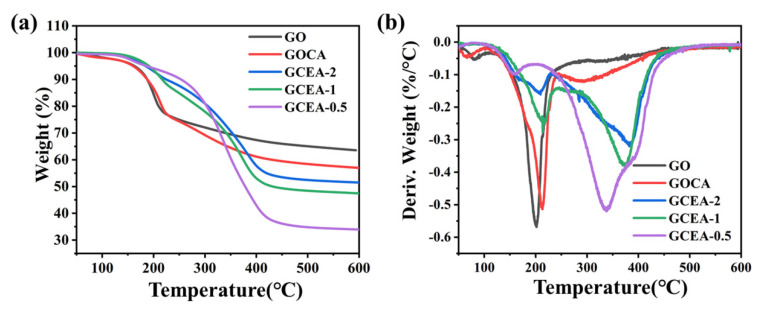
Thermal analysis: (**a**) the TGA curves, and (**b**) the DTG curves (Color figure can be viewed online).

The TGA and DTG curves of the GO, GOCA, and GCEA are presented in Figure 7a,b, respectively. The main weight losses of the GO and GOCA took place at ~150–200 °C, and were attributed to the conversion of the oxygen functionalities to CO and CO_2_, and the evaporation of the absorbed water molecules. By contrast, the GCEA exhibited a distinct decomposition behavior, with a small mass loss at ~200 °C, followed by two major peaks at 215 °C and ~400 °C. The first major peak was attributed to the decomposition of the remaining oxygen functionalities on the GCEA, and was very similar to that of GO and GOCA. However, the weight loss corresponding to the second main peak of GCEA was much greater than that of GO and GOCA, probably due to the breakdown of epoxy groups and GCEA functional groups grafted onto the GO structure. Taken together, the results obtained via FT-IR, Raman, TGA, XPS, and XRD data analysis confirmed the successful preparation of GCEA.

### 2.6. Microwave Absorption Performance 

**Figure 8:**
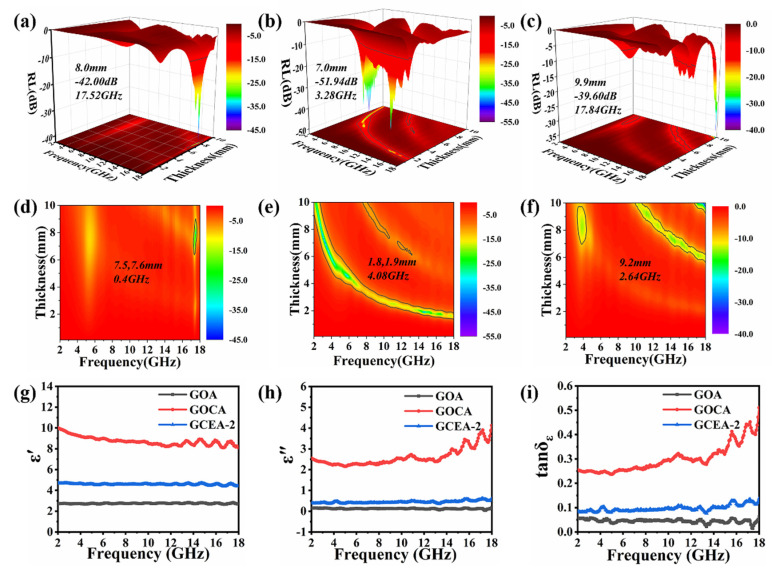
The reflection losses (RL) of the various samples in 2–18 GHz frequency range, (**a**–**c**), 2D plots (**d**–**f**) of GOA, GOCA, GCEA-2 with effective absorption bandwidth. frequency dependence of (**g**) *ε*′, (**h**) *ε*″, (**i**) *tan δ ε* (Color figure can be viewed online).

The MA performance of a material is usually evaluated based on the reflection loss (RL) value. According to the transmission line theory, the detailed RL expression are defined by Equations (1) and (2): [33,34]

(1) $$Z_{in}=Z_{0}\sqrt{\frac{\mu_{r}}{\varepsilon_{r}}}\tan h(j\frac{2\pi fd}{c}\sqrt{\mu_{r}\varepsilon_{r}})$$

(2) $$\mathrm{RL}\left(\mathrm{dB}\right)=20\log\left|\frac{Z_{in}-Z_{0}}{Z_{in}+Z_{0}}\right|$$

where *Z_in_* and *Z*_0_ are the normalized input impedance of the absorber and the impedance of atmospheric space, respectively. *ε_r_* and *μ_r_* are the relative complex permittivity and permeability. *j*, *ƒ*, *d*, as well as *c*, are an imaginary part of the microwave frequency, the specimen thickness, and the velocity of electromagnetic waves in free space. When the RL value is less than −10 dB, it means that 90% of the electromagnetic waves will be absorbed, and the appropriate frequency range can be considered as the effective EAB. At 20% filling ratio, the thickness of GOA, GOCA, and GCEA-2 was 8.1, 7.0, and 9.9 mm, respectively. The 3D plots in Figure 8a–c showed that minimum RL values were −42.0, −51.94, and −35.6 dB at 17.52, 3.28, and 17.84 GHz frequency, respectively. The EAB of GOCA with a thickness of 1.8 mm was 4.08 GHz (Figure 8d). However, the EAB of GCEA was reduced and the RL value was increasesd by a relative decrease in FCNTs content, resulting in significant performance differences (Figure 8e,f, Figures S4 and S5). The introduction of FCNTs increased the dielectric loss and the interfacial polarization, giving samples the highest absorbing properties. 

**Figure 9:**
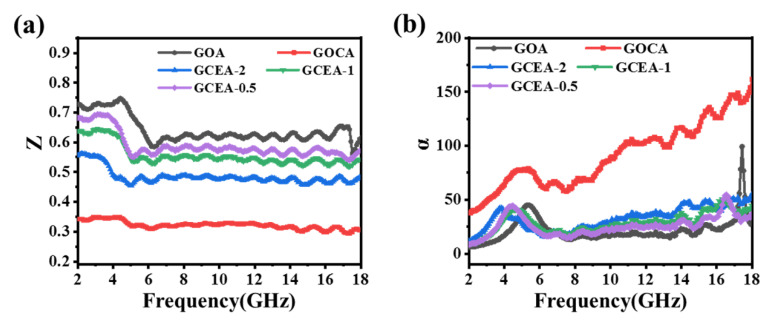
Frequency dependence of (**a**) *Z*, (**b**) *α* for the samples of GOA, GOCA, and GCEA (Color figure can be viewed online).

The EM parameters, complex permittivity (*ε_r_* = *ε*′ − *jε*″), and complex permeability (*μ_r_* = *μ*′ − *jμ*″) are closely related to the microwave absorption performance of composites, where the real parts *ε*′ and *μ*′ indicate the storage capacity of the material for incident microwaves, and the imaginary parts ε″ and *μ*″ indicate the ability to attenuate incident microwaves. It is clear that GOCA has much higher *ε*′, *ε*″, and *tan* *δ ε* of the complex permittivity than that of GO and GCEA-2 in Figure 8g,h. Figure 8i showed that the dielectric loss tangent (*tan δ ε* = *ε*″/*ε*′) of each sample has an obvious relaxation peak between 11 and 18 GHz, suggesting that the interfacial relaxation was responsible for the complex permittivity fluctuations. The redistribution of charge between the FCNTs and GO sheets excited by EM wave initiated the interface relaxation. Furthermore, defects on the graphene and residual oxide-containing groups also led to relaxation. The added FCNTs resulted in better dielectric properties of aerogel [35,36], as evidenced by the Cole–Cole semicircle (*ε*″ versus *ε*′) in Figure S6 (Supporting Information). However, the dielectric constant of the GCEA-2 was decreased (Figure 9i), which was related to the relative reduction of FCNTs in content. Finally, the microwave capture capabilities of the various MA materials are evaluated according to the match in impedance (*Z*), which is equal to |*Z_in_/Z*_0_|. Thus, the closer the Z value is to 1, the better the MA performance [37,38]. The results in Figure 9a indicated that the GO provided the best impedance matching performance, followed by the GCEA-0.5, GCEA-1, GCEA-2, and COCA in decreasing order. In other words, the *Z* value of composite aerogel became closer to 1 as the relative content of FCNTs decreased. Along with the advantages of using aerogels with large specific surface areas and wrinkles as the matrix of the MA material, this approach was expected to provide inspiration for future research on high strength and compressible 3D-absorbing materials.

Additionally, the attenuation constant (*α*), relating to a material’s EM attenuation capability, is calculated using Equation (3): [39]

(3) $$\alpha =\frac{\sqrt{2}\pi f}{c}\times \sqrt{\left(\mu^{\prime \prime }\varepsilon^{\prime \prime }-\mu^{\prime }\varepsilon^{\prime }\right)+\sqrt{{\left(\mu^{\prime \prime }\varepsilon^{\prime \prime }-\mu^{\prime }\varepsilon^{\prime }\right)}^{2}+{\left(\mu^{\prime }\varepsilon^{\prime \prime }+\mu^{\prime \prime }\varepsilon^{\prime }\right)}^{2}}}$$

As can be seen in Figure 9b, GOCA had the highest *α* value, which can be attributed to its higher conductivity. Hence, the introduction of FCNTs led to a higher *α* value for the GOCA compared to the GO. However, a high conductivity can easily lead to a high reflectivity for incident electromagnetic waves, resulting in a poor MA performance [40]. 

**Figure 10:**
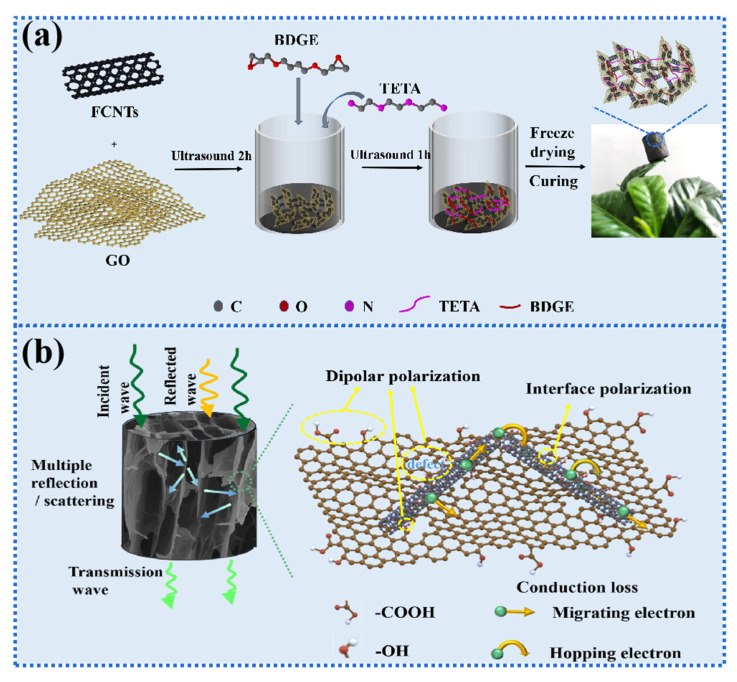
(**a**) Synthesis procedures, (**b**) schematic illustration of EM absorption mechanisms of GCEA composite aerogels (Color figure can be viewed online).

The MA mechanism can be interpreted in Figure 10b: (1) dipole polarization occurs due to the increased numbers of oxygen-containing groups and defects provided by the GO and FCNTs; (2) interfacial polarization occurs due to the difference in electrical conductivity between the interwoven FCNTs and GO; (3) the penetrating 3D interconnected network promoted the leaping of electrons, thus leading to higher conduction and reflection losses [41,42]. Therefore, combined the mechanical strength, structural stability, and MA properties of aerogels, GCEA was sufficient to be applied as a new 3D skeleton in the design of 3D MA materials.

## 3. Conclusions

In summary, double cross-linked graphene oxide aerogels were prepared via a low-cost and simple process, and their skeletons were enhanced by the π-π covalent crosslinking of CNTs and epoxy resin, and by chemical crosslinking. The GCEA exhibited enhanced hydrophilicity and structural stability, and no structural collapse was observed after 30 min of high-frequency ultrasound. The GCEA-2 exhibited super elasticity along with a high energy loss coefficient of 65.08%, and no permanent deformation occurred, even after eight cycles of compression. In addition, the prepared GCEA-2 provided excellent microwave-absorbing properties. In future work, the GCEA will be further reduced or be wrapped by functional particles. Combined with the large specific surface area and wrinkled structure of the aerogel, this formulation is expected to become the ideal skeleton for MA materials.

## 4. Materials and Methods

### 4.1. Materials

Carbon nanotubes (CNTs, 20~40 nm in diameter, purity > 97%, length < 2 μm, special surface area = 70~150 m^3^/g) were obtained from Shenzhen Nano Company (China). Functionalized carbon nanotubes (FCNTs) were manufactured from carbon nanotubes by Institute of Polymer Materials, Qingdao University. Graphite powder, Ethanol, 1,4-butanediol diglycidyl ether (BDGE), and triethylenetetetramine (TETA) were purchased from Aladdin Reagent Company, Shanghai, China. Sulphuric acid (H_2_SO_4_), nitric acid (HNO_3_), hydrochloric acid (HCl), hydrogen peroxide (H_2_O_2_), and potassium permanganate (KMnO_4_) were purchased from Sinopharm Chemical Reagent Co. All reagents are of analytical grade. 

### 4.2. Preparation of GO

Graphite oxide was prepared from natural graphite by modified Hummers method [43]. Next, 8 g graphite, 3.75 g NaNO_3_, and 360 mL H_2_SO_4_ (98%) were added in a three-necked flask and mechanically stirred in ice water bath for 30 min for pre-oxidation. Next, 22.5 g potassium permanganate was slowly added to the above mixture, being stirred in an ice water bath for another 2 h. After that, the above mixture was heated to 35 °C for 17 h, then 400 mL of deionized water was slowly added to it and stirred for 1 h. Subsequently, 660 mL deionized water and 60 mL hydrogen peroxide were poured into the above mixture for 20 min. Afterwards, the mixture was sonicated for 30 min and then washed with dilute hydrochloric acid until the mixture supernatant did not precipitate when added to 0.1mol/L BaCl_2_ solution. Finally, the GO suspension was neutralized to pH 7 against deionized water. 

### 4.3. Preparation of FCNTs

Functionalized carbon nanotubes (FCNTs) were manufactured from carbon nanotubes (CNTs) [44,45]. 750 mL of concentrated H_2_SO_4_ and 250 mL of HNO_3_ were put into a three-necked flask containing 8 g of CNTs to stir with ultrasonic for 15 min, and then reflux at 100 °C for 8 h. After the reaction, the mixture was washed with deionized water until the pH was 7, and then functionalized carbon nanotubes (FCNTs) were obtained by freeze-drying.

### 4.4. Preparation of GCEA

In our previous work, we found that the aerogel structure had the most uniform structure and the best mechanical properties when the GO to FCNTs mass ratio was 7:3 [13]. Therefore, samples with GO/FCNTs to epoxy resin mass ratios of 3:1, 2:1, 1:1, 1:2, and 1:3 (recorded as GCEA-3, GCEA-2, GCEA-1, GCEA-0.5, and GCEA-0.33) were prepared on this basis. FCNTs were stripped in deionized water to obtain FCNTs dispersion with the assistance of ultrasound. Next, GO suspension was added to FCNTs dispersion and ultrasound for 2 h. BDGE, TETA, and C_2_H_5_OH were added to the above mixture and ultrasonically again for 2 h. The mixture was then placed in a bottle and frozen in liquid nitrogen, followed by vacuum freeze-drying for 3 days. Finally, the samples were obtained after the oven at 100 °C for 6h. Sample composition of GCEA is shown in Table 1, and the preparation process is shown in Figure 10a.

### 4.5. Characterization

The structure and elemental composition of aerogels was analyzed by x-ray diffraction (XRD, Ultima IV with Cu-Kα radiation, λ = 1.5418 Å, Rigaku Corporation, Matsubaracho, Japan). Fourier infrared spectrometer (FT-IR, Nicolet is50, Thermo Scientific, Waltham, MA, USA) and Raman spectroscopy (Invia RM2000, Renishaw plc, Gloucestershire, UK) were used to identify functional groups species and structures, respectively. In addition, X-ray photoelectron spectroscopy (XPS, ESCALAB 220i-XL, Thermo Fisher, Vlastimila Pecha, Czech Republic) is employed to further demonstrate the chemical elemental composition of aerogels. The morphology and internal microstructure of aerogels were observed by field emission scanning electron microscope (SEM, JSM-78OOF, JEOL, Matsubaracho, Japan) and transmission electron microscope (TEM, 2100Plus, JEOL, Matsubaracho, Japan). A universal material testing machine (Instron 5300, Instron, Canton, OH, USA) was employed to research the mechanical properties of aerogels with a compression rate of 1 mm/min and a compression range of 0–100 N. Thermal gravimetric analysis (TGA) was analyzed using an SDT650 thermal analyzer (TA, Brookville, PA, USA) in nitrogen at 10 °C/min in the range of 38–700 °C. Powder specimens were mixed with solid paraffin wax in a given proportion by mass and pressed into hollow ring-shaped test specimens with an external diameter of 7 mm, an internal diameter of 3.04 mm, and a height of 2–3 mm, and a vector network analyzer (N5234A, (NYSE: A, Palo Alto, USA) was employed to obtain the EM parameters, including the relative complex permittivity (*ε_r_* = *ε*′ − *jε*″) and relative complex permeability (*μ_r_* = *μ*′ − *jμ*″) in the 2.0–18.0 GHz frequency range with a coaxial test. MATLAB was used to calculate the reflection loss.

## Figures and Tables

**Table 1:** Sample composition.

Sample	GO/(12.63 mg/mL)/mL	FCNTs/mg	BDGE/mg	TETA/mg	H_2_O/mL	Ethanol
GOCA	16	84	0	0	12	2.2
GCEA-3	16	84	75.39	18.12	12	2.2
GCEA-2	16	84	112.82	27.18	12	2.2
GCEA-1	16	84	225.64	54.36	12	2.2
GCEA-0.5	16	84	451.26	108.74	12	2.2
GCEA-0.33	16	84	676.89	163.11	12	2.2

## Data Availability

Not applicable.

## Supplementary Materials

The following supporting information can be downloaded at: https://www.mdpi.com/article/10.3390/gels8100618/s1, Figure S1: XRD pattern (a,b) and Raman spectroscopy (c,d) of GCEAs; Figure S2: Compressive stress-strain cyclic curves of (a) GCEA-0.33, (b) GCEA-3. Figure S3: compressive stress-strain cyclic curves of GCEA-0.5, GCEA-1 at strain of 40% for 7 cycles. Figure S4: Frequency dependence of (a) *ε*′, (b) *ε*″, (c) *tanδε* for the samples of GCEA-1, GCEA-0.5; Figure S5: (a,b) reflection loss plots of GCEA-0.5, GCEA-1, Figure S6: *ε*′ − *ε*″ plot of GO (a), FCNTs (b), GOCA (c), GCEA-2 (d), GCEA-1 (e), GCEA-0.5 (f). References [46,47] are cited in the supplementary materials.
