# Supplementary material for: Structurally Stable, High-Strength Graphene Oxide/Carbon Nanotube/Epoxy Resin Aerogels as Three-Dimensional Skeletal Precursors for Wave-Absorbing Materials
Source: Gels. 2022 Sep 28;8(10):618. doi: 10.3390/gels8100618 (PMC9601949; doi:10.3390/gels8100618)
Supplement: Supplementary file 1 [file gels-08-00618-s001.zip › gels-1916220-supplementary.pdf]

Structurally Stable, High-Strength Graphene Oxide/Carbon Nanotube/Epoxy Resin Aerogels as Three-Dimensional Skeletal Precursors for Wave-Absorbing Materials

Lina Zhang, Guojun Song, Zetian Zhao, Lichun Ma, Hui Xu, Guanglei Wu, Yinghu Song, Yinuo Liu, Lihan Qiu, Xiaoru Li*

Institute of Polymer Materials, School of Material Science and Engineering, Qingdao University, Qingdao 266071, China

* Corresponding author e-mail address: lixiaoruqdu@126.com (X. Li)

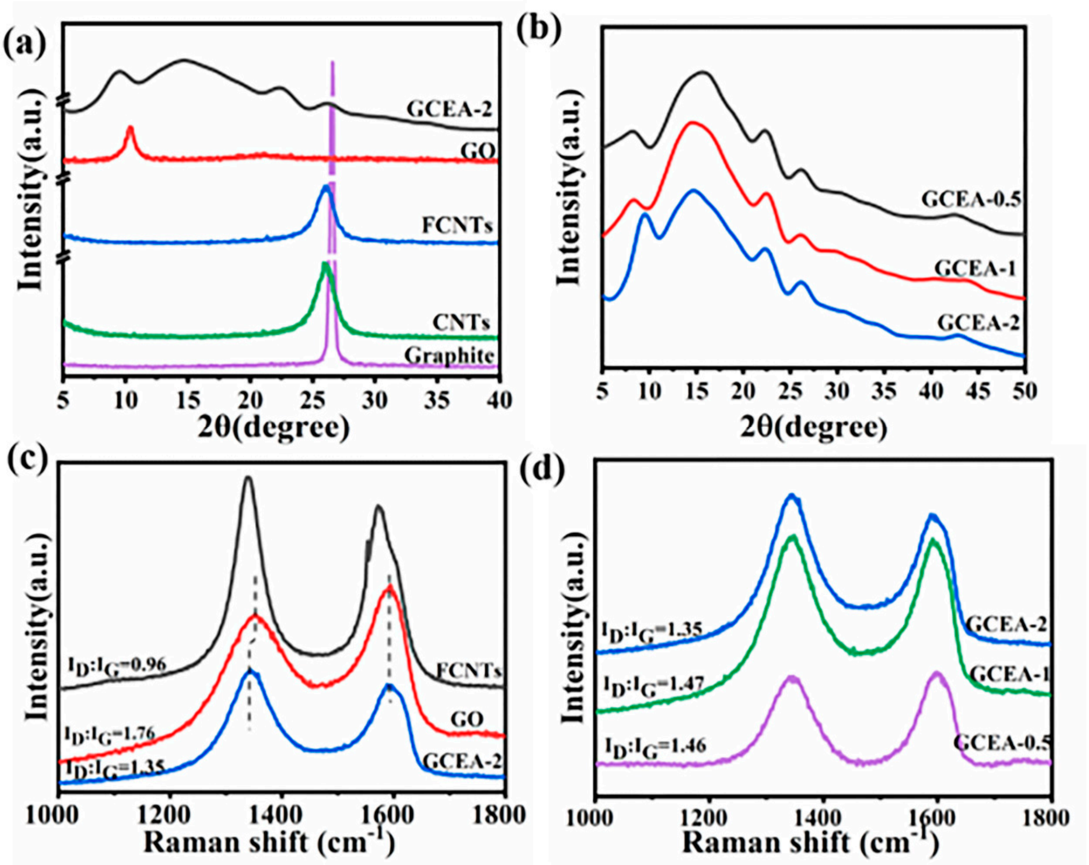

Fig. S1. XRD pattern (a,b) and Raman spectroscopy (c,d) of GCEA.

Figure S1a and b presented the XRD of the samples. The diffraction peaks of FCNTs and CNTs were located at 25.95°, indicating that oxidation has no effect on the interlayer spacing of the CNTs. The diffraction peak of the graphite powder at 26.5° was attributed to the reflection of the graphite (002) surface, corresponding to a layer spacing of 0.34 nm, which disappeared after the graphite is oxidized, and the (001) diffraction peak corresponding to a layer spacing of 0.86 nm appear at about 10.4°, demonstrating the successful introduction of hydroxyl and carboxyl groups and the successful exfoliation of the graphite in the chemical reaction. the diffraction peak representing GO (001) plane in GCEA-2 was transferred to 9.5°, corresponding to the interlayer spacing of 9.3 nm. Furthermore, the (001) diffraction peak of GO shifted to the left as the epoxy content increases, which indicated that the layer spacing becomes broad, seen from Fig. S1b. Therefore, TETA was covalently bonded onto the GO layer to achieve broad layer spacing. The peak of GCEA around 26° belong to the typical

peak of FCNTs, and the broad peak in the 14-24.9° range may relate to epoxy resin. The amorphous state of XRD of GCEA may be caused by epoxy resin covering GO.

The Raman data also provided evidence for successful GO grafting as shown in Figure S1(c, d). As we all know, the D band is related to the defects in the sp^3 hybrid carbon bond, and the characteristic peak of the G band corresponds to the first-order scattering of the E_{2g} mode observed for the sp^2 carbon domains, which is a typical peak of sp^2 hybrid graphitic carbon[1]. A ratio of intensities (I_D/I_G) in the D and G bands can intuitively reflect the degree of defects of carbon materials. When epoxy was introduced into GOCA, D-band shift of GCEA-2 from 1354 cm^{-1} to 1341 $cm^{-1,}$ indicating a strong interaction between GO and TETA[2]. Moreover, the I_D/I_G ratio of graphene oxide decreased from 1.76 to 1.35, indicating that graphene oxide had more defects, and with the grafting molecule replacing some sp3 carbon sites of graphene oxide and generating more sp^2 carbon forms, which made the defects repaired[3]. With the increase of crosslinking agent content, I_D / I_G increased slightly, which may be due to epoxy resin covered on graphene oxide sheets, resulting in increased disorder.

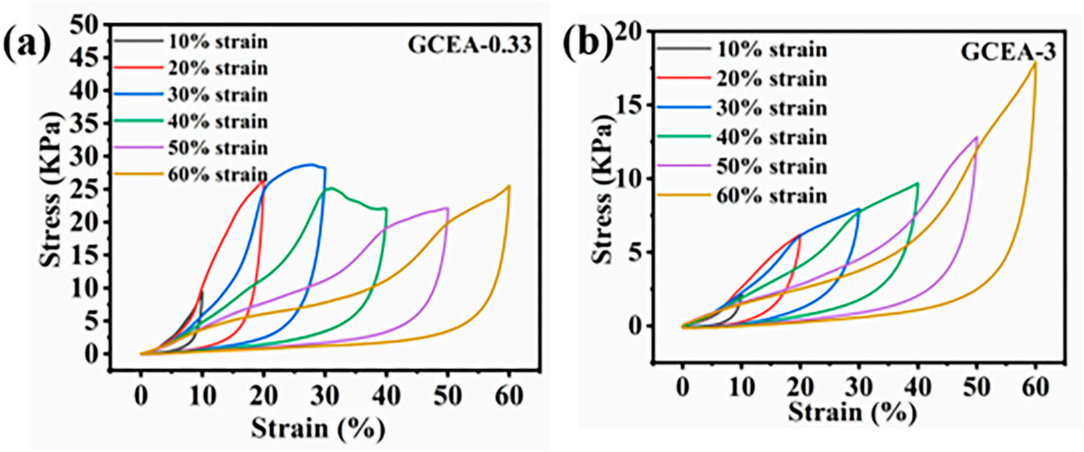

Figure S2. Compressive stress-strain cyclic curves of (a) GCEA-0.33, (b) GCEA-3.

As shown in Fig. S2, when the content of epoxy resin increases, the brittleness of GCEA-0.33 increased, which was not conducive to the later compounding with polymer. When GCEA-3 was compressed by 50%, its stress decreased at 40% displacement, so it was considered that its resilience was worse than that of GCEA-2. Therefore, this manuscript only discussed the mass ratio of GO/FCNTs to the epoxy resin of 2:1, 1:1, 1:2 respectively for the following work.

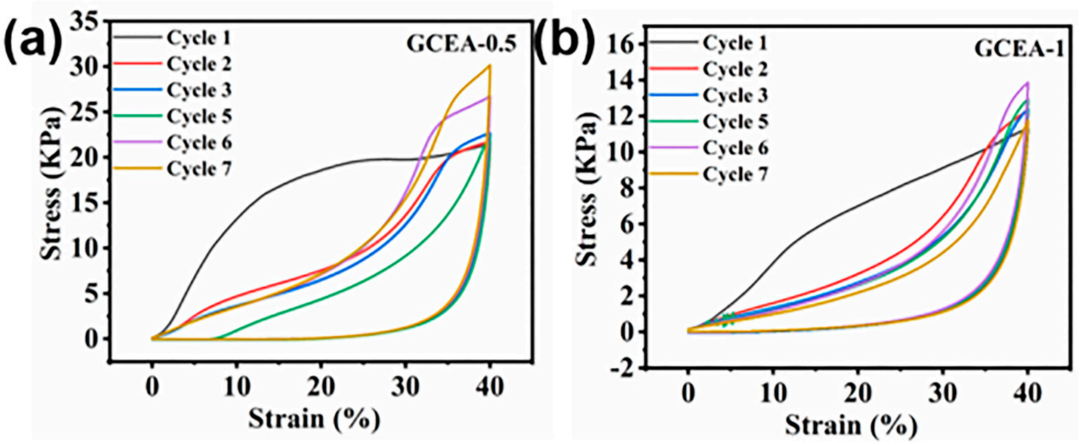

Figure S3. Compressive stress-strain cyclic curves of GCEA-0.5, GCEA-1 at strain of 40% for 7 cycles.

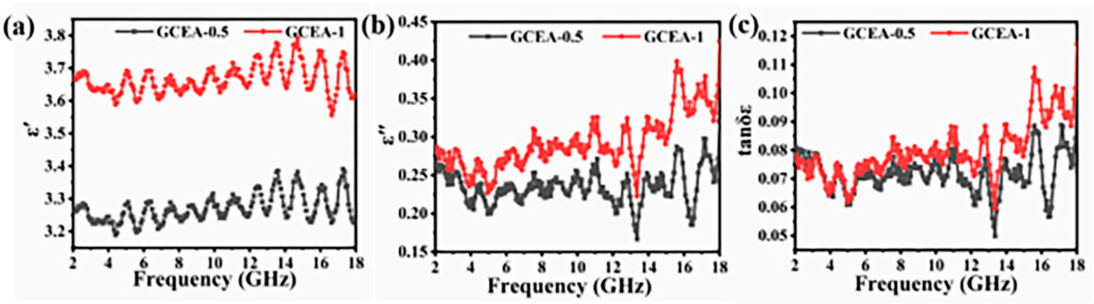

Figure S4. Frequency dependence of (a)ε', (b)ε'', (c) $tan\delta\varepsilon$ for the samples of GCEA-1, GCEA-0.5.

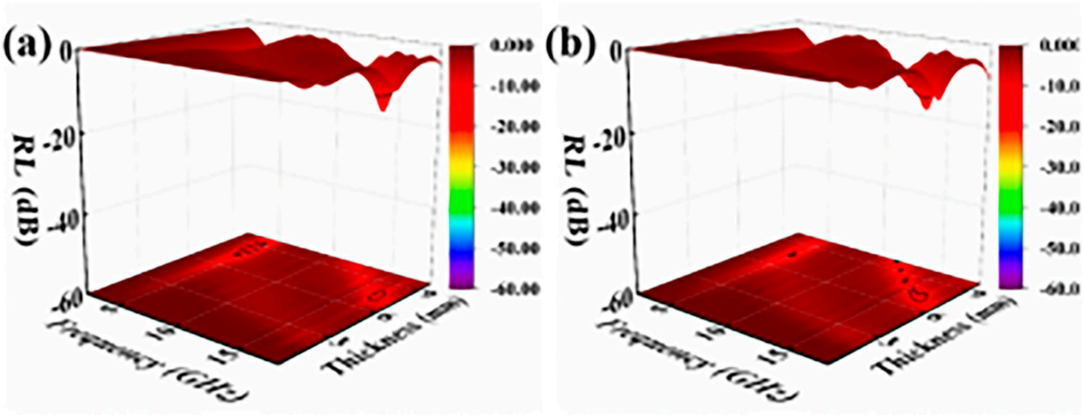

Figure S5. (a, b) reflection loss plots of GCEA-0.5, GCEA-1.

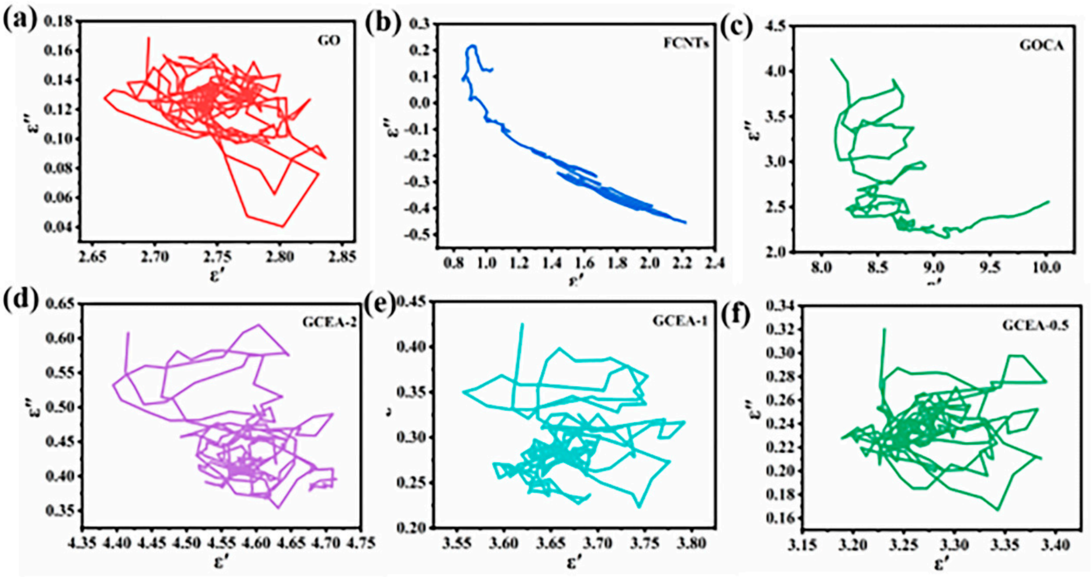

Figure S6. $\varepsilon'-\varepsilon''$ plot of GO(a), FCNTs(b), GOCA(c), GCEA-2(d), GCEA-1 (e), GCEA-0.5 (f).

Generally speaking, the complex dielectric constant was mainly affected by the

Debye relaxation process, and the Debye model can explain the process of dielectric relaxation, the description of the Cole-Cole semicircle can be deduced from the following equation[4]:

$$(\varepsilon' - \frac{\varepsilon_s + \varepsilon_\infty}{2})^2 + (\varepsilon'')^2 = (\frac{\varepsilon_s - \varepsilon_\infty}{2})^2$$

ε_s and ε_∞ are the static permittivity and the relative permittivity in the high frequency range, respectively. According to the above equations, it is found that the ε' and ε'' curves can form a semicircle, and each semicircle represents a sequential Debye relaxation process[5]. As shown in Fig. S6, GOCA contains multiple polarization relaxation and conduction losses thanks to the synergistic effect of GO and FCNTs. Nevertheless, the GCEA (Fig. S6 d-f) curves are complicated, confirming the existence of multiple mechanisms of polarization within the GCEA.

References

[1] Song, G.; Gai, L.; Yang, K.; Wang, X.; An, Q.; Xiao, Z.; Zhai, S. A versatile N-doped honeycomb-like carbonaceous aerogels loaded with bimetallic sulfide and oxide for superior electromagnetic wave absorption and supercapacitor applications. *Carbon* **2021**, *181*, 335-347. doi:http://dx.doi.org/10.1016/j.carbon.2021.05.044.

[2] Zhou, T.; Zhang, J.; Zhao, J.; Qu, W.; Li, X.; Li, S.; Xing, B.; Fu, Y. In-situ grafted graphene oxide-based waterborne epoxy curing agent for reinforcement corrosion protection of waterborne epoxy coating. *Surf. Coat. Technol.* **2021**, *412*, 127043. doi:http://dx.doi.org/https://doi.org/10.1016/j.surfcoat.2021.127043.

[3] Song, B.; Sizemore, C.; Li, L.; Huang, X.; Lin, Z.; Moon, K.-s.; Wong, C.-P. Triethanolamine functionalized graphene-based composites for high performance supercapacitors. *J. Mater. Chem. A* **2015**, *3*, 21789-21796. doi:http://dx.doi.org/10.1039/c5ta05674h.

[4] Liang, L.; Li, Q.; Yan, X.; Feng, Y.; Wang, Y.; Zhang, H.B.; Zhou, X.; Liu, C.; Shen, C.; Xie, X. Multifunctional Magnetic Ti3C2Tx MXene/Graphene Aerogel with Superior Electromagnetic Wave Absorption Performance. *ACS Nano* **2021**, *15*, 6622-6632. doi:http://dx.doi.org/10.1021/acsnano.0c09982.

[5] Xu, X.; Wang, G.; Wan, G.; Shi, S.; Hao, C.; Tang, Y.; Wang, G. Magnetic Ni/graphene connected with conductive carbon nano-onions or nanotubes by atomic layer deposition for lightweight and low-frequency microwave absorption. *Chem. Eng. J.* **2020**, *382*, 122980. doi:http://dx.doi.org/https://doi.org/10.1016/j.cej.2019.122980.
